# Laser-induced pinpoint hydrogen evolution from benzene and water using metal free single-walled carbon nanotubes with high quantum yields†
†Electronic supplementary information (ESI) available: GC and HPLC analyses for products characterisation (Fig. S1–S3 and S5), time course data of H_2_ evolution in various solvents (S4), IR (S6), TG (S7) and experimental details (S8). See DOI: 10.1039/c4sc02269f



**DOI:** 10.1039/c4sc02269f

**Published:** 2014-09-09

**Authors:** Kei Ohkubo, Naoki Kohno, Yusuke Yamada, Shunichi Fukuzumi

**Affiliations:** a Department of Material and Life Science , Graduate School of Engineering , Osaka University , ALCA , Japan Science and Technology Agency (JST) , Suita , Osaka 565-0871 , Japan . Email: fukuzumi@chem.eng.osaka-u.ac.jp ; Fax: +81 6 6879 7370 ; Tel: +81 6 6879 7368

## Abstract

Metal-free photocatalytic hydrogen evolution occurred efficiently in benzene containing single-walled carbon nanotubes under laser irradiation at 532 nm with an extremely high turnover number of 2 000 000 and a high quantum yield of 130%. The rate of hydrogen evolution increased with increasing laser intensity to exhibit a fourth power dependence, suggesting that hydrogen was evolved *via* four-photon processes in which the coupling of two radical anions derived from benzene is the rate-determining step and the benzene radical anion is produced by electron transfer from benzene to the doubly excited state of single-walled carbon nanotubes, which requires two photons. Polymerisation of benzene was induced by the photogenerated C_6_H_6_˙^–^, accompanied by hydrogen evolution, resulting in a leverage effect to increase the quantum yield of hydrogen evolution to well over the 25% expected for the four-photon process. Laser-induced hydrogen evolution also occurred in water containing single-walled carbon nanotubes. In contrast to the case of benzene, water was not oxidized but hydrogen evolution from water was accompanied by the multi-oxidation of single-walled carbon nanotubes. The yield of hydrogen based on one mole of single-walled carbon nanotubes with 1.4 nm diameter and 1–5 mm length was determined to be 2 700 000%, when oxidations of single-walled carbon nanotubes occurred to produce the polyhydroxylated product.

## Abstract 2

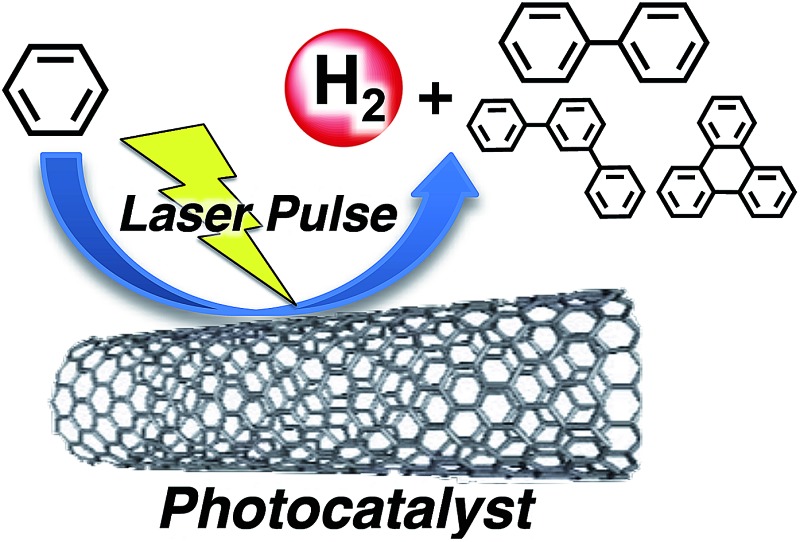
Metal-free photocatalytic hydrogen evolution occurred efficiently in benzene containing SWCNTs under laser irradiation with an extremely high turnover number of 2 000 000 and a high quantum yield of 130%.

## Introduction

Carbon nanomaterials have been widely studied for their potential applications as electrode materials for efficient energy conversion and storage.^1–5^ Metal-free nanostructured elemental carbons and carbon-based composites have proven to be attractive alternatives to conventional metal-based catalysts for several important chemical reactions such as dehydrogenation reactions of aromatic compounds,^5–7^ oxygenation^8^ and Friedel–Crafts reactions.^9,10^ However, these reactions were carried out under severe conditions to activate substrate molecules.

In particular, single-walled carbon nanotubes (SWCNTs) have been of great interest to researchers because of their unique structures and physical properties.^11–17^ SWCNTs have been proposed as advanced metal-catalyst supports for electrochemical catalysis.^18–20^ However, there are no reports that SWCNTs alone are used as photocatalysts under ambient conditions due to their poor photochemical and excited properties.

Catalytic hydrogen (H_2_) evolution systems have been extensively studied because hydrogen is a clean energy source for the future, which should reduce dependence on fossil fuels and emissions of greenhouse gases in the long term.^21,22^ In many cases, noble metals such as platinum and semiconductors have been used as photocatalysts.^23–35^ However, there are no reports on photocatalytic metal-free H_2_ evolution systems using pure carbon alone as a photocatalyst.^36^

We report herein efficient H_2_ evolution from benzene and benzene derivatives using metal free SWCNTs alone as a photocatalyst under visible laser light irradiation (532 nm) at room temperature and atmospheric pressure with a high quantum yield of 130%. Efficient laser-induced hydrogen evolution was also observed from water with SWCNTs. The reaction mechanisms of laser-induced H_2_ evolution from benzene and water with SWCNTs are clarified based on the oxidized products, deuterium kinetic isotope effects and the dependence of the rate of H_2_ evolution on the laser intensity. This is the first example of laser-induced H_2_ evolution with high quantum yields, paving a new way for pinpoint H_2_ production using a laser pulse, which may find various applications.

## Results and discussion

### Laser-induced photocatalytic hydrogen evolution from benzene with SWCNTs

**Fig. 1:**
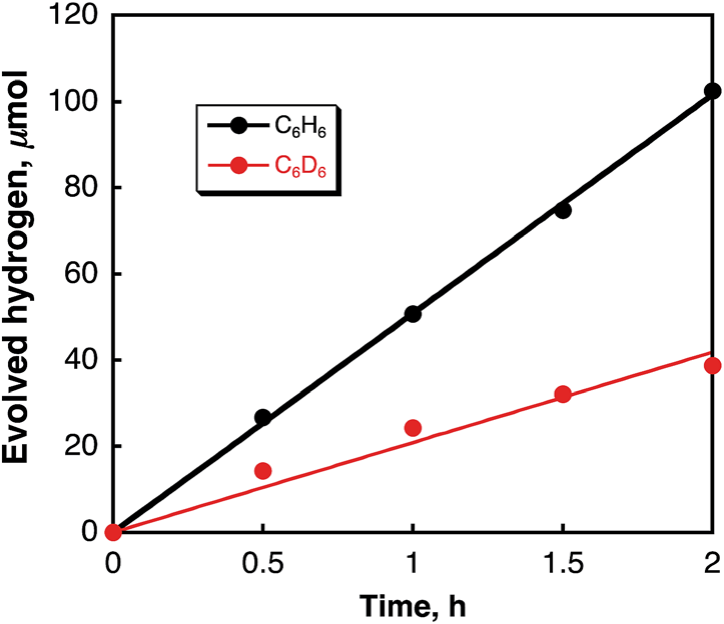
Time courses of hydrogen evolution in deaerated C_6_H_6_ (black circles) and C_6_D_6_ (red circles) containing SWCNTs (0.060 mg mL^–1^) under laser irradiation at 532 nm (50 mJ per pulse).

Laser pulse irradiation (*λ* = 532 nm; 500 mW; 10 Hz) of a deaerated benzene solution (2.5 mL) containing dispersed metal-free SWCNTs (0.15 mg) resulted in efficient hydrogen evolution. The amount of hydrogen evolved by laser irradiation for 2 h reached 100 μmol (2.2 mL). The quantum yield of hydrogen evolution was determined from the initial rate to be 34%. When benzene was replaced by deuterated benzene (C_6_D_6_), the deuterated hydrogen molecules such as D_2_ and HD were also evolved efficiently (see Fig. S1 in the ESI†). The initial rate of hydrogen evolution was 21 μmol h^–1^ with a 50 : 1 ratio for D_2_ and HD, respectively. The KIE (kinetic isotope effect) value was determined from the slopes in Fig. 1 to be 2.4. These results indicate that the hydrogen source is benzene. The products derived from the dehydrogenation of benzene were determined by GC-MS and HPLC analyses to be biphenyl, terphenyls, and terphenylene (see Fig. S2 and S3 in ESI†). The stoichiometry required to produce H_2_ and biphenyl from benzene is given by eqn (1).1
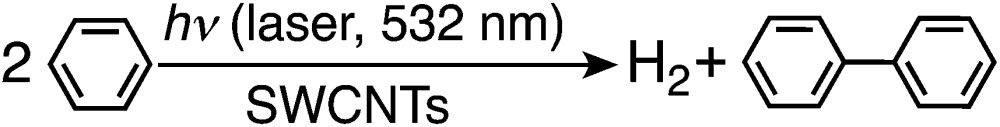



**Fig. 2:**
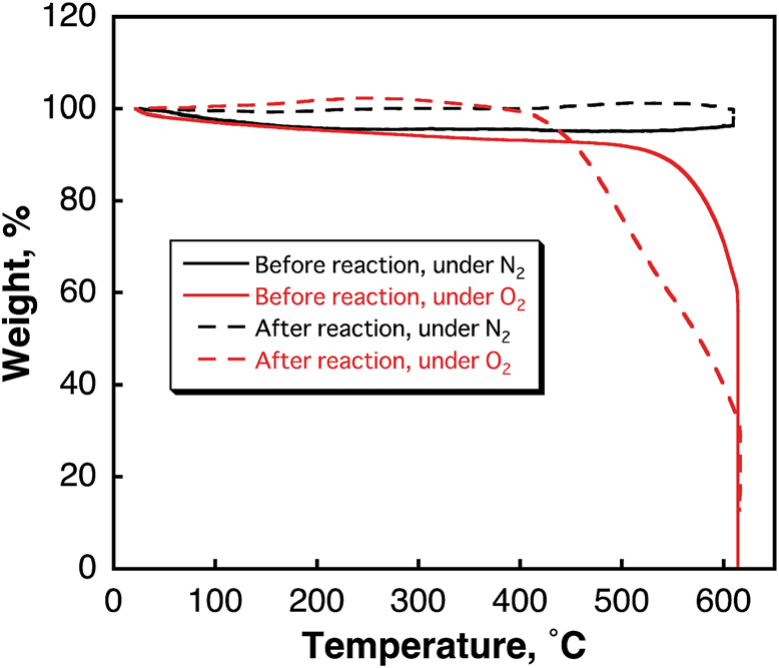
TG curves of SWCNTs before and after laser light irradiation (H_2_ evolution) in benzene observed under deaerated and aerated conditions.

There is no evidence for the functionalisation of SWCNTs by benzene under photoirradiation, which was observed by TG analyses (Fig. 2) because no weight loss from the decomposition of functionalized molecules to SWCNTs was observed at low temperature. Thus, hydrogen was evolved *via* the condensation reaction of benzene in the photocatalytic reaction.

Hydrogen was also evolved in various aromatic solvents with electron withdrawing and donating substituent(s) (Fig. S4 in ESI†). The amounts of H_2_ evolved are listed in Table 1. The highest catalytic activity was obtained in benzene. The catalytic turnover number (TON) is roughly estimated as (2.0 ± 0.5) × 10^6^ per SWCNT for 2 h irradiation, calculated from the tube diameter and average length of the SWCNTs with a zig–zag structure used in this study.^37^ When benzene was replaced by benzene-*d*_6_, the TON decreased to (8.0 ± 2.0) × 10^5^ per SWCNT. This value agrees with the KIE of 2.4 determined from the initial rate of H_2_ evolution as shown in Fig. 1.

**Table 1:** Amount of H_2_ evolved in various solvents after laser irradiation for 2 h

Solvent	H_2_ evolved^*a*^ μmol
Benzene	100
Mesitylene	71
Toluene	66
*p*-Xylene	60
Chlorobenzene	54
Benzonitrile	39
1,2-Dimethoxybenzene	12

^*a*^Conditions: SWCNTs (0.15 mg) dispersed in deaerated solvent (2.5 mL). Excited at 532 nm (50 mJ per pulse).

**Fig. 3:**
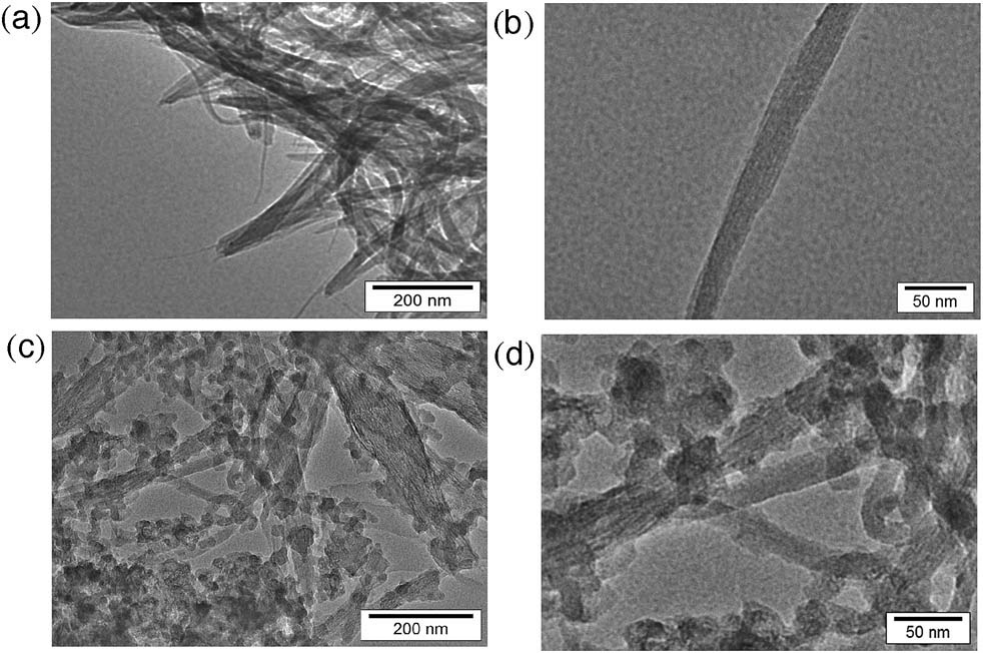
TEM images of SWCNTs (a and b) before and (c and d) after laser photoirradiation (50 mJ per pulse, 10 Hz) for 2 h in deaerated benzene at 298 K.

Transmission electron microscopy (TEM) measurements were performed to evaluate the transformation of SWCNTs before and after hydrogen evolution. The TEM images before photoirradiation (Fig. 3a and b) clearly exhibit tubular morphologies. There are no inorganic impurities in the commercially available and highly purified SWCNTs used in this study (see Experimental section). After hydrogen evolution, the tubular structure was partially changed to an agnail structure and small clusters. However, a tubular component still remains (Fig. 3c and d).

**Fig. 4:**
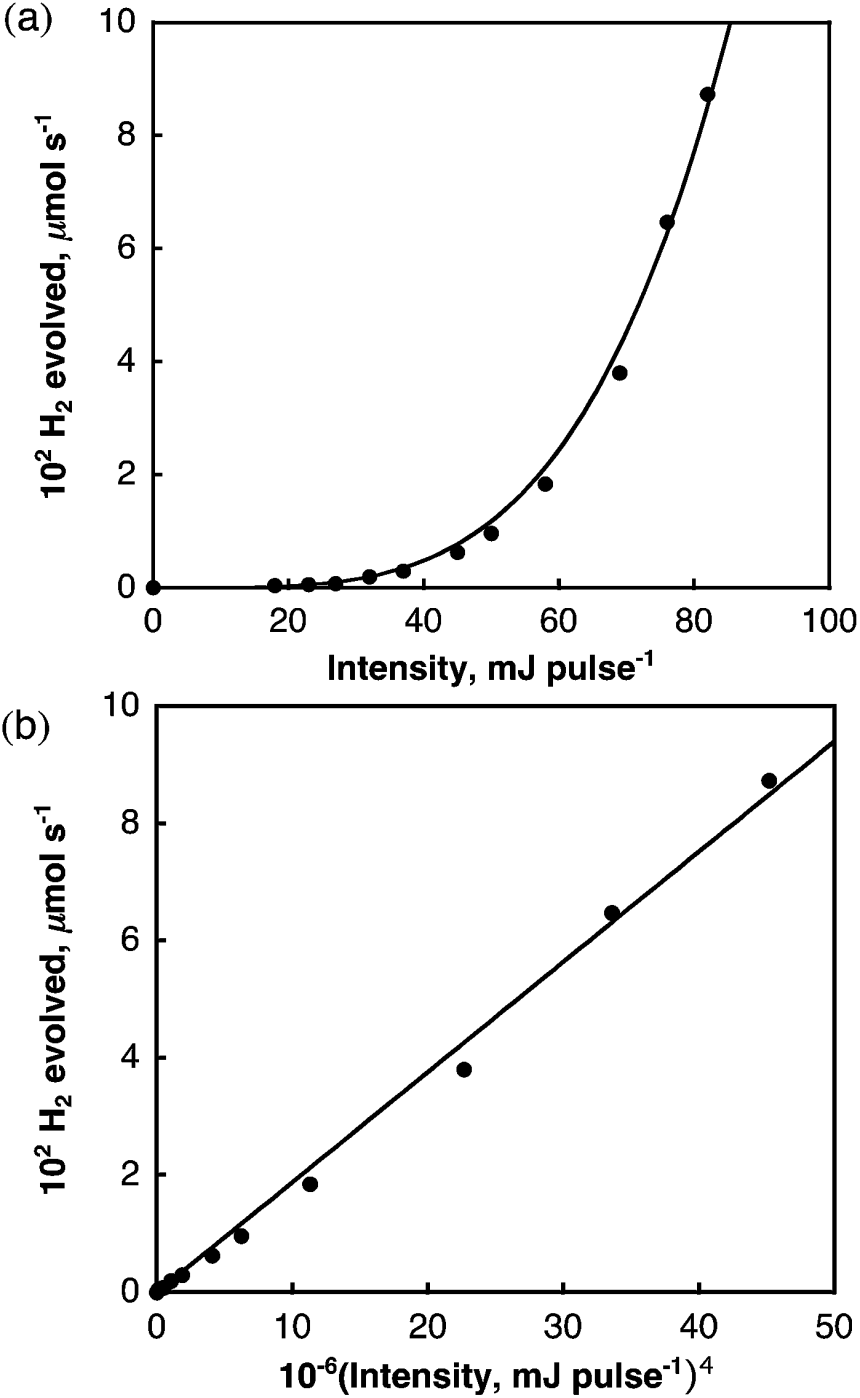
(a) Plot of initial rate of H_2_ evolved in deaerated benzene *vs.* laser power intensity at 532 nm; (b) plot of initial rate of H_2_ evolved *vs.* the fourth power of the laser intensity.

The dependence of the rate of H_2_ evolution on the laser intensity was examined using different laser power intensities at 532 nm (0–82 mJ per pulse). The initial rates of H_2_ evolution are proportional to the fourth power of the laser intensity as shown in Fig. 4. This suggests that a bimolecular reaction of two-photon absorbed species may be involved in the photocatalytic hydrogen evolution.

**Scheme 1:**
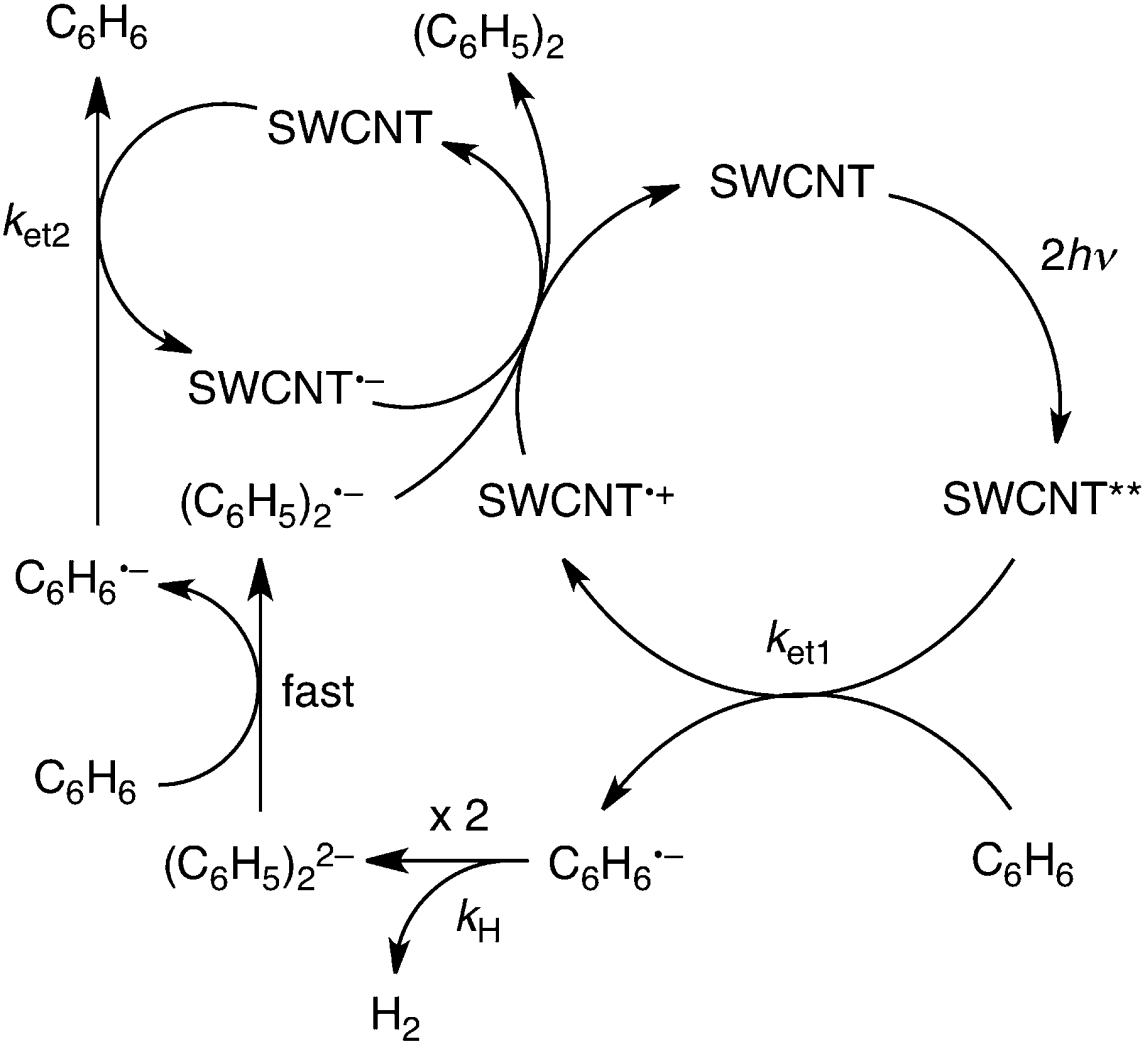


On the basis of the above-mentioned results, the plausible photocatalytic mechanism for hydrogen evolution in benzene is shown in Scheme 1. A SWCNT was excited by two photons to form a doubly excited SWCNT (SWCNT**). The SWCNT** can reduce benzene to produce the benzene radical anion (C_6_H_6_˙^–^) and the one-electron oxidized SWCNT (SWCNT˙^+^) with the rate constant *k*_et1_. Dimerisation of C_6_H_6_˙^–^ occurs, accompanied by hydrogen evolution to produce the biphenyl dianion [(C_6_H_5_)_2_^2–^] with the rate constant *k*_H_. Such hydrogen evolution *via* the radical coupling of C_6_H_6_˙^–^ has previously been reported for the reduction of benzene with a cesium nano carbon catalyst.^38^ (C_6_H_5_)_2_^2–^ can reduce benzene to produce C_6_H_6_˙^–^ and the biphenyl radical anion [(C_6_H_5_)_2_˙^–^] in benzene.^39^ Back electron transfer from (C_6_H_5_)_2_˙^–^ to SWCNT˙^+^ results in the formation of biphenyl [(C_6_H_5_)_2_], accompanied by the regeneration of the SWCNT. On the other hand, C_6_H_6_˙^–^ can reduce the SWCNT to produce SWCNT˙^–^ and C_6_H_6_ with the rate constant *k*_et2_. The charge recombination from SWCNT˙^–^ to SWCNT˙^+^ also regenerates the SWCNT. The overall stoichiometry of the photocatalytic cycle in Scheme 1 agrees with eqn (1).

According to Scheme 1, the rate of hydrogen evolution is given by eqn (2). The rate of formation and decay of C_6_H_6_˙^–^ is given by eqn (3).

2 d[H_2_]/d*t* = *k*_H_[C_6_H_6_˙^–^]^2^

3 d[C_6_H_6_˙^–^]/d*t* = *k*_et1_[SWCNT**] – *k*_H_[C_6_H_6_˙^–^]^2^ – *k*_et2_[SWCNT][C_6_H_6_˙^–^]

Assuming that *k*_et2_[SWCNT][C_6_H_6_˙^–^] ≫ *k*_H_[C_6_H_6_˙^–^]^2^, the steady-state concentration of C_6_H_6_˙^–^ is given by eqn (4).

4 [C_6_H_6_˙^–^] = *k*_et1_[SWCNT**]/(*k*_et2_[SWCNT])

From eqn (2) and (4), the rate of hydrogen evolution is rewritten by eqn (5).

5 d[H_2_]/d*t* = *k*_H_ [*k*_et1_[SWCNT**]/(*k*_et2_[SWCNT])]^2^

Because the concentration of SWCNT** is proportional to the square of the laser intensity, the rate of hydrogen evolution is proportional to the fourth power of the laser intensity as observed in Fig. 4b. The observed deuterium kinetic isotope effect on the photocatalytic H_2_ evolution in Fig. 1 suggests that the C–H bond cleavage of C_6_H_6_˙^–^ (*k*_H_) is involved as the rate-determining step in the radical coupling for H_2_ evolution in Scheme 1.

**Scheme 2:**
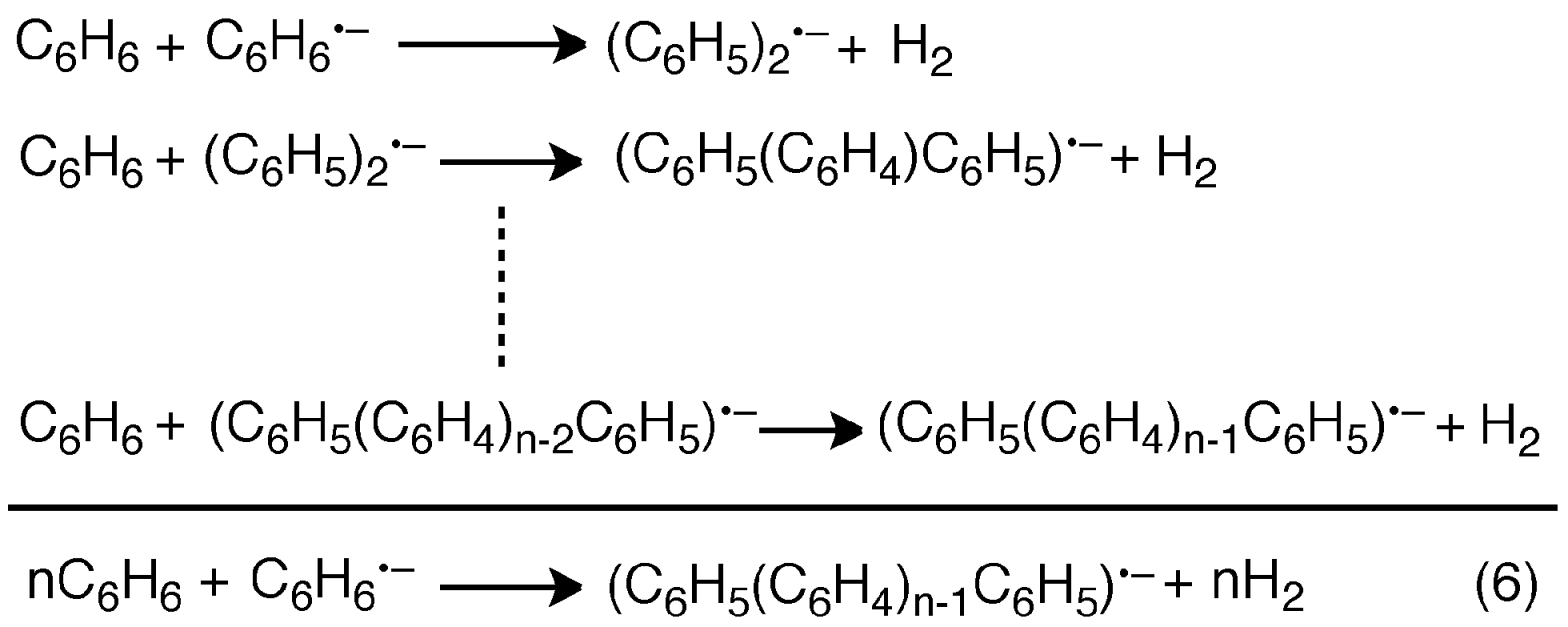


In Scheme 1, C_6_H_6_˙^–^ produced by the electron transfer from C_6_H_6_ to SWCNT** dimerizes to afford H_2_ and (C_6_H_5_)_2_^2–^, followed by rapid electron transfer from C_6_H_6_ to (C_6_H_5_)_2_^2–^ to yield C_6_H_6_˙^–^ and (C_6_H_5_)_2_˙^–^. The overall reaction of C_6_H_6_˙^–^ with C_6_H_6_ to yield H_2_ and (C_6_H_5_)_2_˙^–^ is shown in Scheme 2, where (C_6_H_5_)_2_˙^–^ can react further with C_6_H_6_ to produce the terphenyl radical anion [(C_6_H_5_(C_6_H_4_)C_6_H_5_)˙^–^] and H_2_. The same type of reaction continues and the overall reaction is given by eqn (6). Thus, once one mol of C_6_H_6_˙^–^ is produced, *n* moles of hydrogen can be produced from *n* moles of benzene to form the radical anion of a benzene polymer [(C_6_H_5_(C_6_H_4_)_*n*–1_C_6_H_5_)˙^–^], which may undergo charge recombination with SWCNT˙^+^ to regenerate the SWCNT.

According to Scheme 1, biphenyl is produced by the four-photon process when the maximum value of the quantum yield is 25%. However, the polymerisation of benzene induced by photo-generated C_6_H_6_˙^–^ in Scheme 2 gains a leverage effect to increase the quantum yield of H_2_ evolution above that expected from the four-photon process. Indeed, the highest quantum yield of H_2_ evolution was determined to be 130% at a laser power of 82 mJ per pulse under the conditions in Fig. 2a, where the photon number of the laser pulse was calibrated by ferrioxalate actinometry (see the experimental section in ESI†).^40^

The efficiency of the photocatalytic H_2_ evolution is affected by substitution of the benzene ring with electron donating or withdrawing substituents (Table 1). The efficiency of photocatalytic H_2_ evolution is highest for benzene and lowest for 1,2-dimethoxybenzene, because substitution on the benzene ring may retard the radical coupling with hydrogen and the electron donating substituents (methoxy group) may slow down electron transfer to the SWCNT** in Scheme 1.

**Fig. 5:**
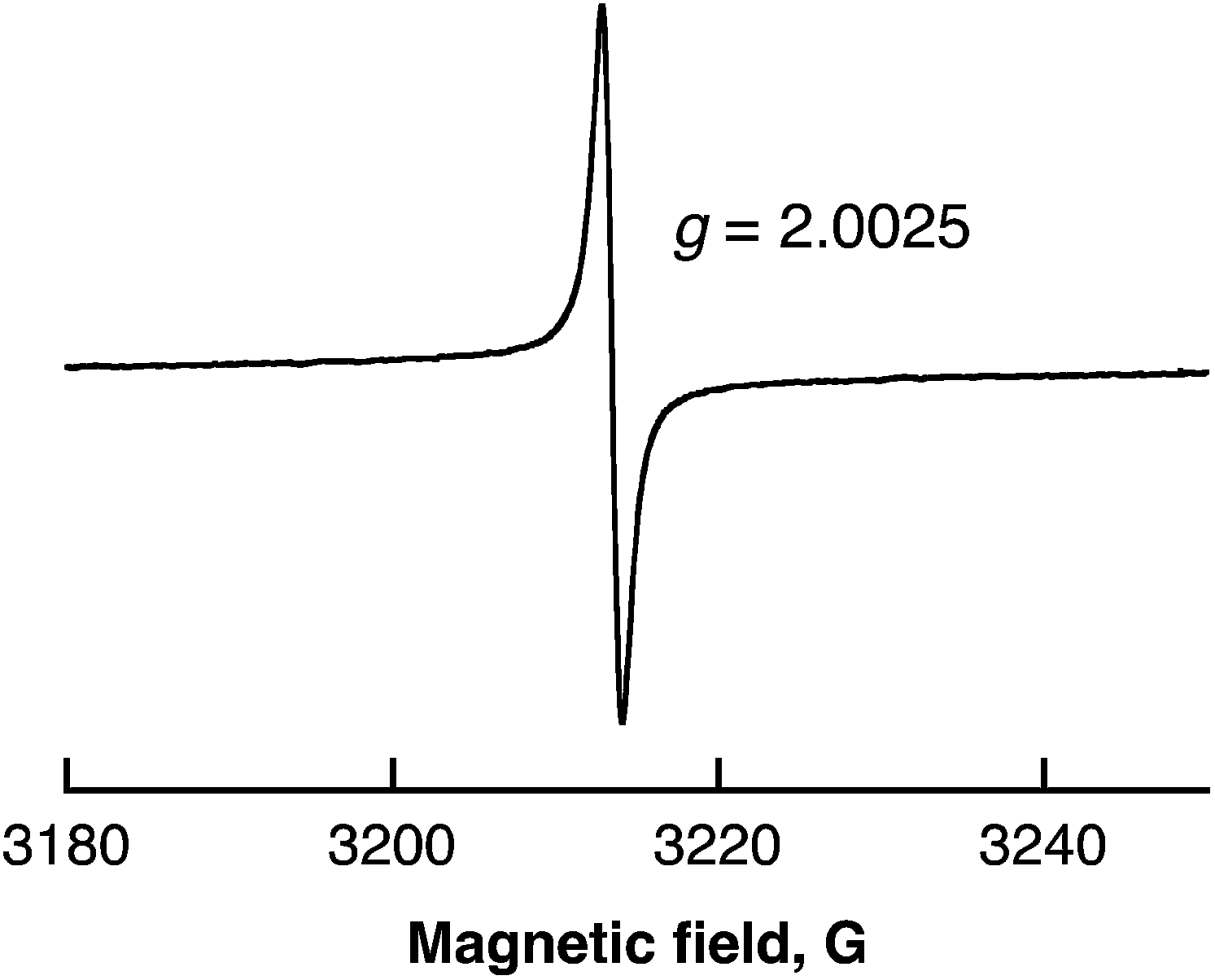
ESR spectrum of benzene containing SWCNTs (0.12 mg mL^–1^) after laser pulse irradiation at 532 nm (40 mJ per pulse; 10 Hz) for 10 min at 77 K.

Radical intermediates involved in the photocatalytic H_2_ evolution from benzene with SWCNTs were detected by ESR spectroscopy measured at 77 K as shown in Fig. 5. The observed ESR signal in the region of *g* = 2.0025 can be assigned to radical anions derived from benzene,^41^ which are overwrapped with SWCNT˙^+^.^42^ The intensity of the ESR signal increased with increasing photoirradiation time because the stability of radical anions is expected to increase as the polymerisation of benzene in Scheme 2 proceeds.

### Laser-induced hydrogen evolution from water with SWCNTs

**Fig. 6:**
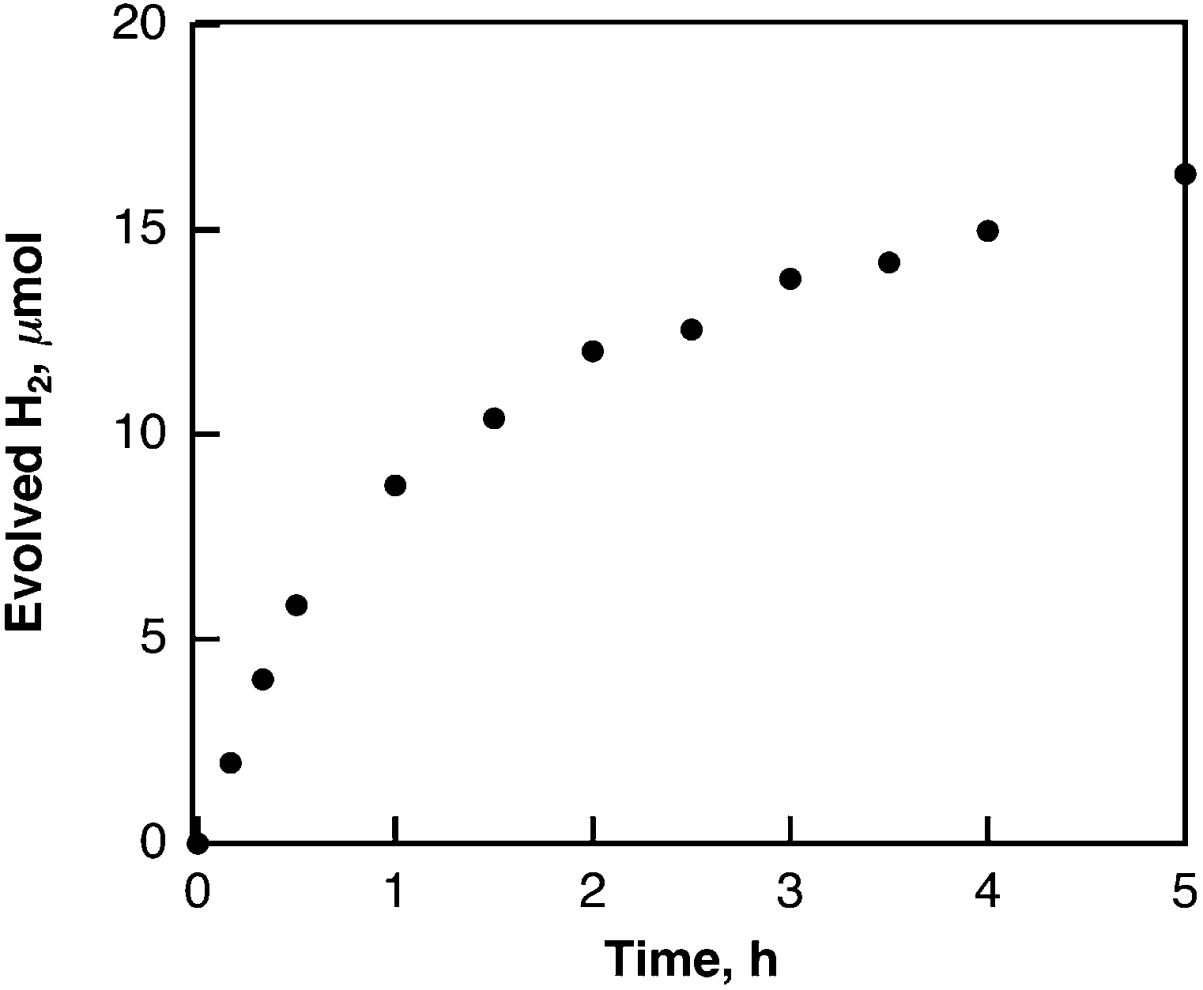
Time course of hydrogen evolution in deaerated H_2_O containing SWCNTs (0.80 mg mL^–1^) under laser irradiation at 532 nm (60 mJ per pulse; 10 Hz).

**Fig. 7:**
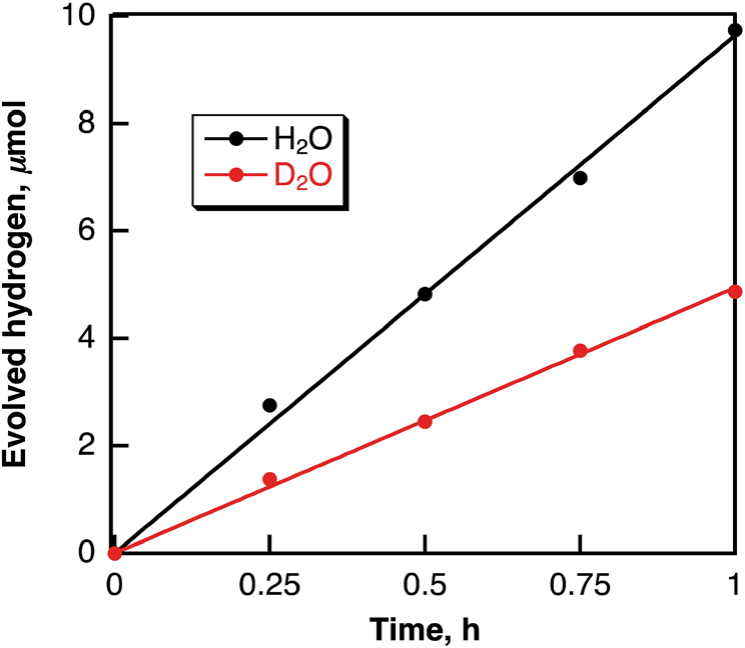
Time courses of H_2_ and D_2_ evolution in deaerated H_2_O (black circles) and D_2_O (red circles), respectively, containing SWCNTs (0.80 mg mL^–1^) under laser irradiation at 532 nm (60 mJ per pulse; 10 Hz).

Hydrogen evolution also occurred in deaerated H_2_O (2.5 mL) containing dispersed metal-free SWCNTs (2.0 mg) under Nd–YAG laser pulse irradiation (*λ* = 532 nm; 600 mW; 10 Hz) as shown in Fig. 6. The amount of evolved H_2_ after 5 h reached 16.4 μmol, which is 2.7 × 10^4^ times larger than the amount of SWCNT (0.6 nmol) calculated from the tube diameter and average length of SWCNTs with a zig–zag structure used in this study. When H_2_O was replaced by D_2_O, the deuterated hydrogen molecules such as D_2_ and HD were also evolved (see Fig. S5 in ESI†) and the KIE value was determined from the ratio of the H_2_ evolution in H_2_O *vs.* D_2_O (Fig. 7) to be 1.9, which is somewhat smaller than the value in benzene. These results indicate that the hydrogen source of evolved H_2_ is water.

**Fig. 8:**
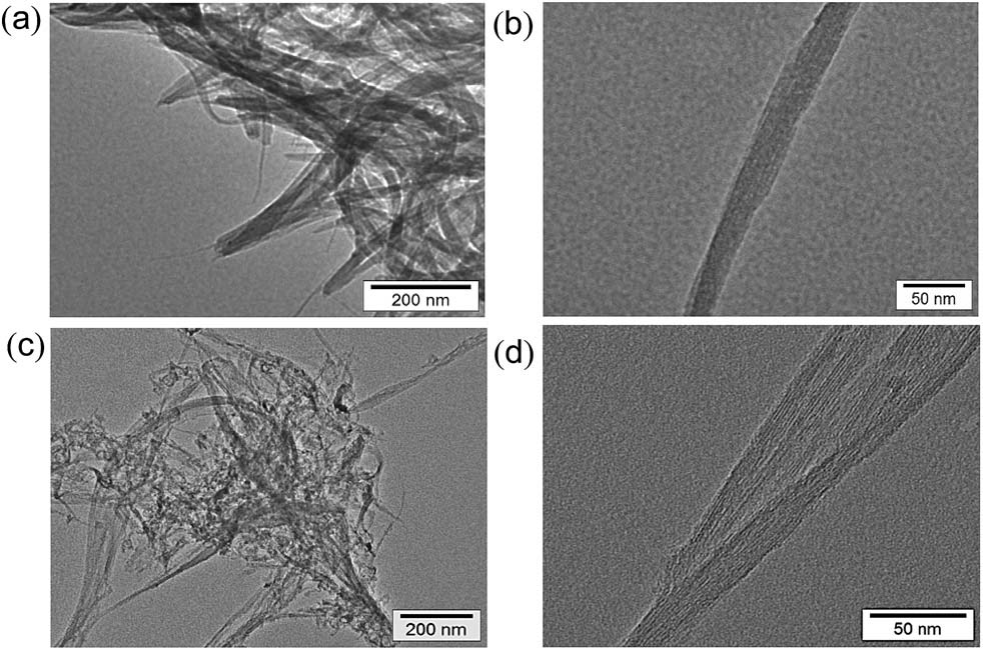
TEM images of SWCNTs (a, b) before and (c, d) after laser photoirradiation (60 mJ per pulse, 10 Hz) for 2 h in deaerated H_2_O at 298 K.

**Fig. 9:**
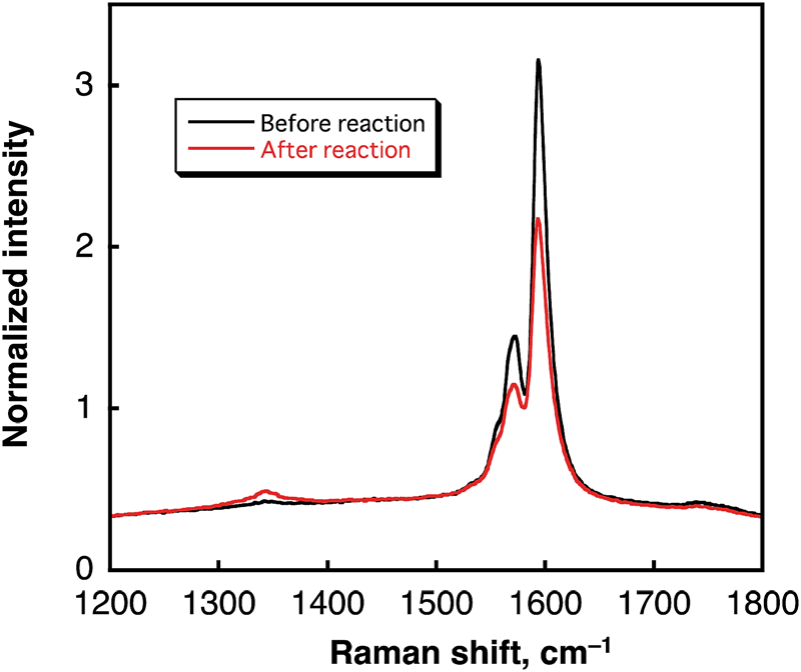
Raman spectra of SWCNTs before laser irradiation (black line) and (b) SWCNTs obtained by laser light irradiation (*λ* = 532 nm; 60 mJ per pulse; 10 Hz) for 5 h in deaerated water (0.80 mg mL^–1^, red line).

In contrast to the case of benzene, no oxidized form of water (dioxygen or hydrogen peroxide) was produced after H_2_ evolution (see Fig. S3 in ESI†). In such a case, SWCNTs may be oxidized, accompanied by the laser-induced H_2_ evolution. Comparison of the TEM images of SWCNTs before and after laser photoirradiation in H_2_O (Fig. 8) indicates that the tubular morphology remained after the H_2_ evolution by laser photoirradiation. Comparison of the Raman spectra of SWCNTs before and after laser photoirradiation of SWCNTs dispersed in deaerated H_2_O are shown in Fig. 9. The G-bands at 1590 and 1570 cm^–1^ decreased with the appearance of the D-band at 1340 cm^–1^. The increased D/G ratio observed after the photoirradiation suggests that sidewall functionalisation of SWCNTs occurred.^43,44^ The IR spectra also suggest that SWCNTs were hydroxylated to exhibit O–H stretching vibrations at 3200–3600 cm^–1^ (Fig. S6 in ESI†).

Comparison of the weight loss of SWCNTs before and after the laser irradiation in H_2_O observed in the TG measurements (Fig. S7 in ESI†) also suggests that SWCNTs were hydroxylated. The number of OH groups of one SWCNT is estimated from the elemental analyses of SWCNTs before (C 96.81% and H 0.19%)^45^ and after evolution of 16.4 μmol of H_2_ (C 91.88% and H 0.60%) to be 18 000. Thus, the H_2_ evolution is accompanied by the two-electron oxidation of a SWCNT (attachment of two OH groups). The stoichiometry of the laser-induced H_2_ evolution with SWCNTs in H_2_O is given by eqn (7).7
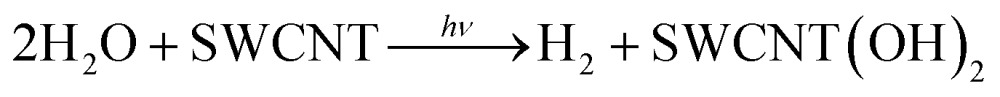



**Fig. 10:**
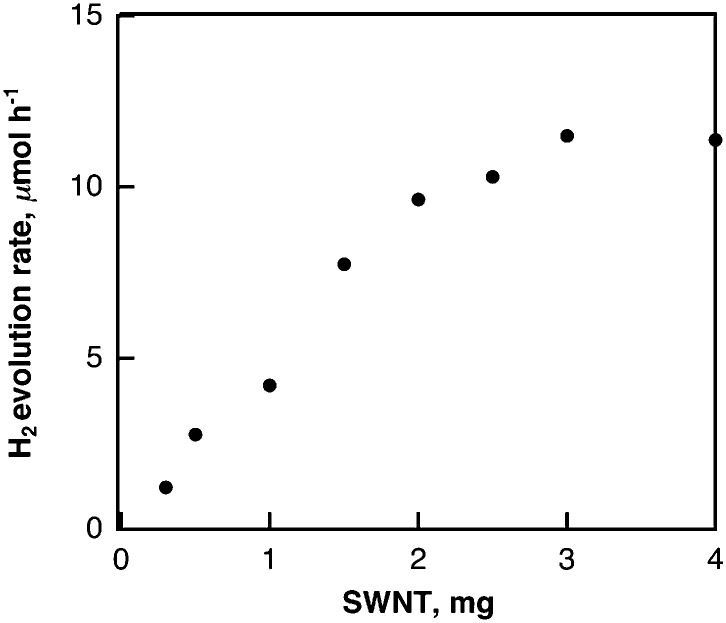
Plot of rate of H_2_ evolution in deaerated H_2_O (2.5 mL) containing various amounts of SWCNTs under laser irradiation at 532 nm (60 mJ per pulse; 10 Hz) *vs.* amount of SWCNTs.

**Fig. 11:**
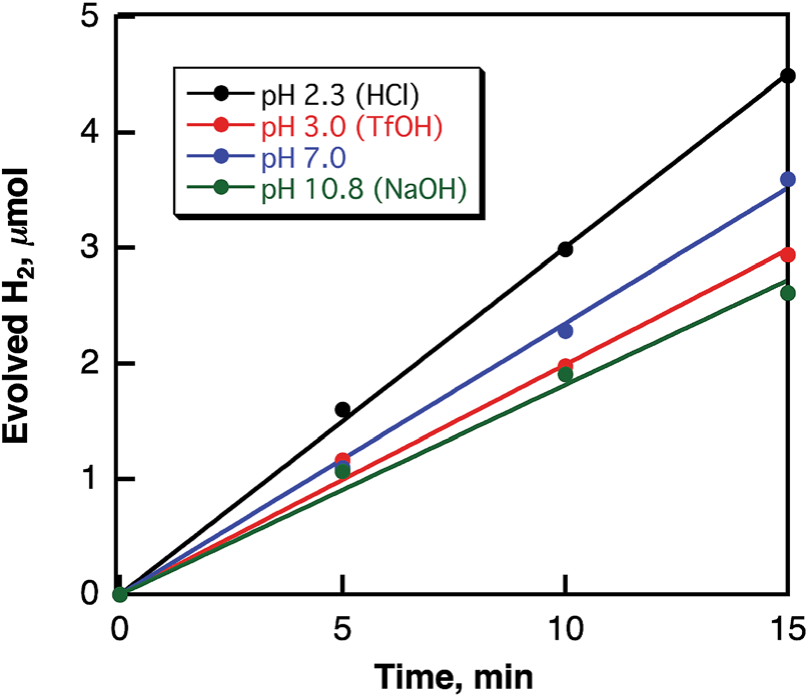
Time courses of H_2_ evolution in deaerated H_2_O containing various amounts of SWCNTs (0.80 mg mL^–1^) under laser irradiation at 532 nm (60 mJ per pulse; 10 Hz) at various pHs.

The laser-induced H_2_ evolution rate with SWCNTs in H_2_O increases with the increasing amount of SWCNTs to reach a constant value as shown in Fig. 10. This shows sharp contrast with the case of the laser-induced H_2_ evolution with SWCNTs in benzene, when the rate of H_2_ evolution was independent of the amount of SWCNTs as discussed above based on Scheme 1. The pH dependence of the H_2_ evolution rate was also examined as shown in Fig. 11, where the rate of H_2_ evolution is rather independent of pH.

**Fig. 12:**
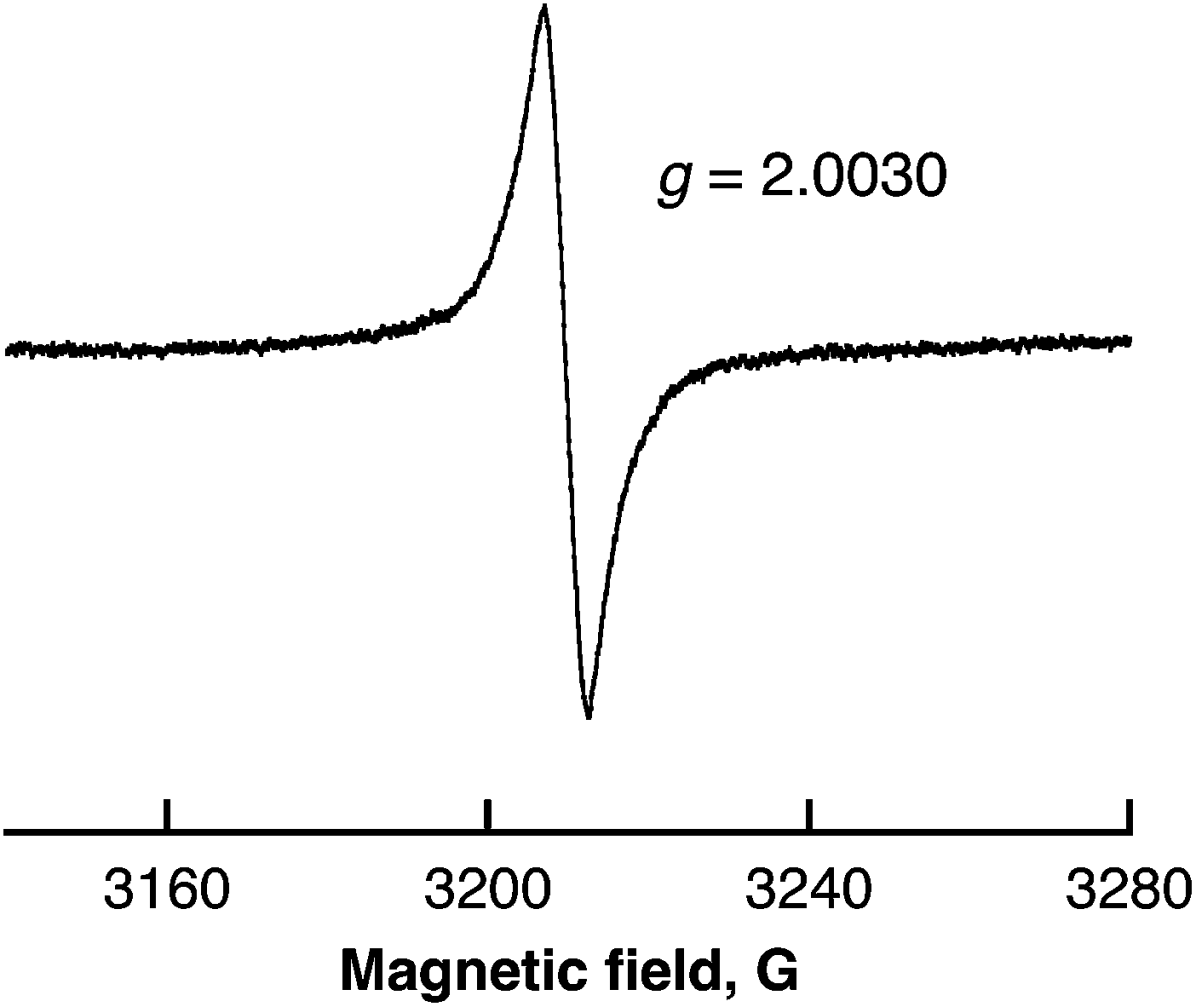
ESR spectrum of frozen water containing SWCNTs (0.80 mg mL^–1^) after laser pulse irradiation at 532 nm (60 mJ per pulse; 10 Hz) for 10 min at 77 K.

The overall stoichiometry agrees with that in eqn (7). The EPR spectrum exhibits a radical intermediate in the H_2_ evolution with SWCNTs from water as shown in Fig. 12. The EPR signal was clearly observed at *g* = 2.0030 under laser irradiation at 77 K in frozen water containing SWCNTs. The *g* value is larger than the signal at *g* = 2.0025 observed in benzene (Fig. 5). Such a large *g* value indicates the existence of SWCNT(OH)˙ as a radical intermediate due to the spin–orbit coupling of oxygen in the laser-induced H_2_ evolution with SWCNTs in water.

**Fig. 13:**
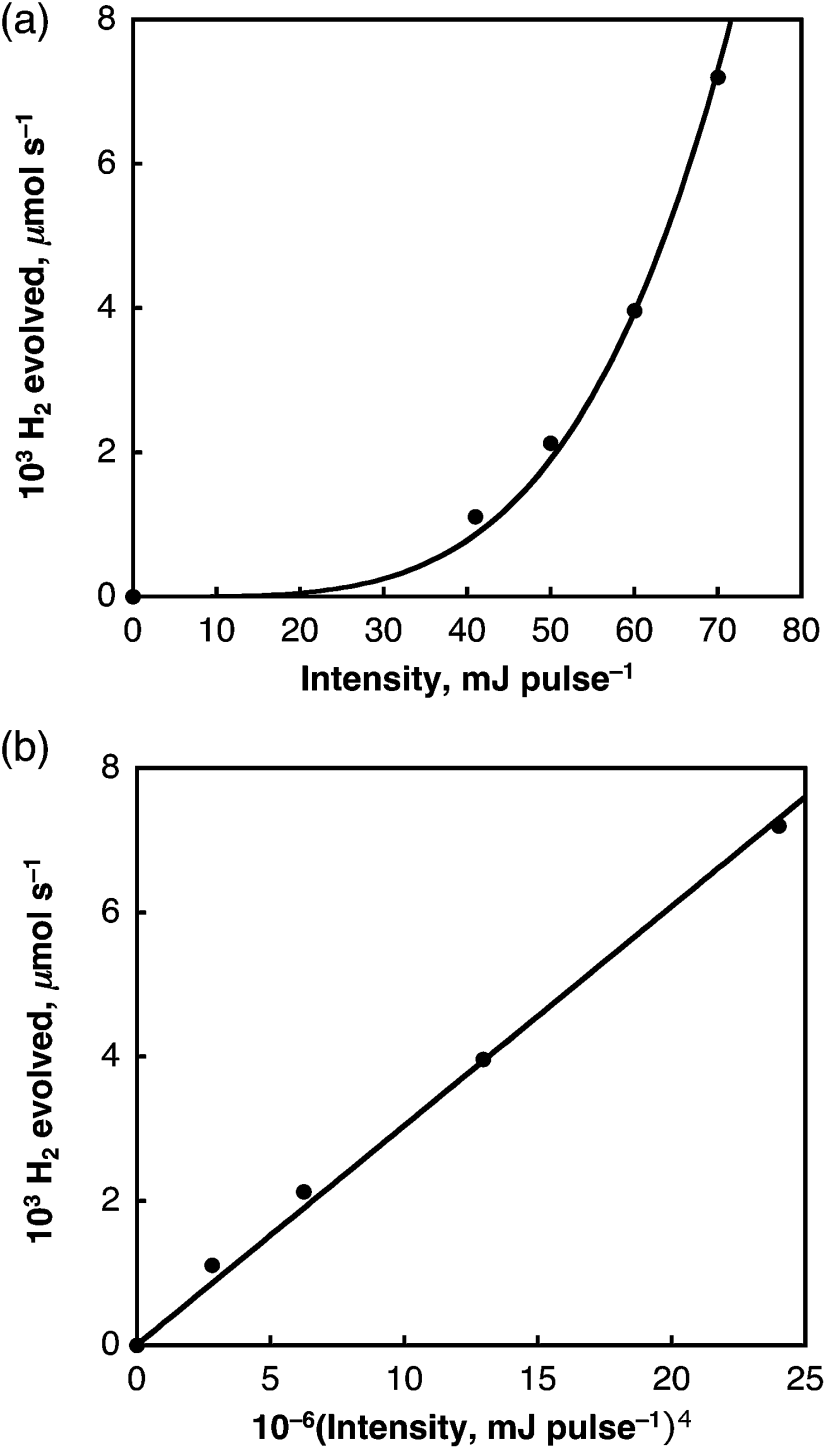
(a) Plot of initial rate of H_2_ evolved in H_2_O *vs.* laser power intensity at 532 nm; (b) plot of initial rate of H_2_ evolved *vs.* the fourth power of laser intensity.

**Scheme 3:**
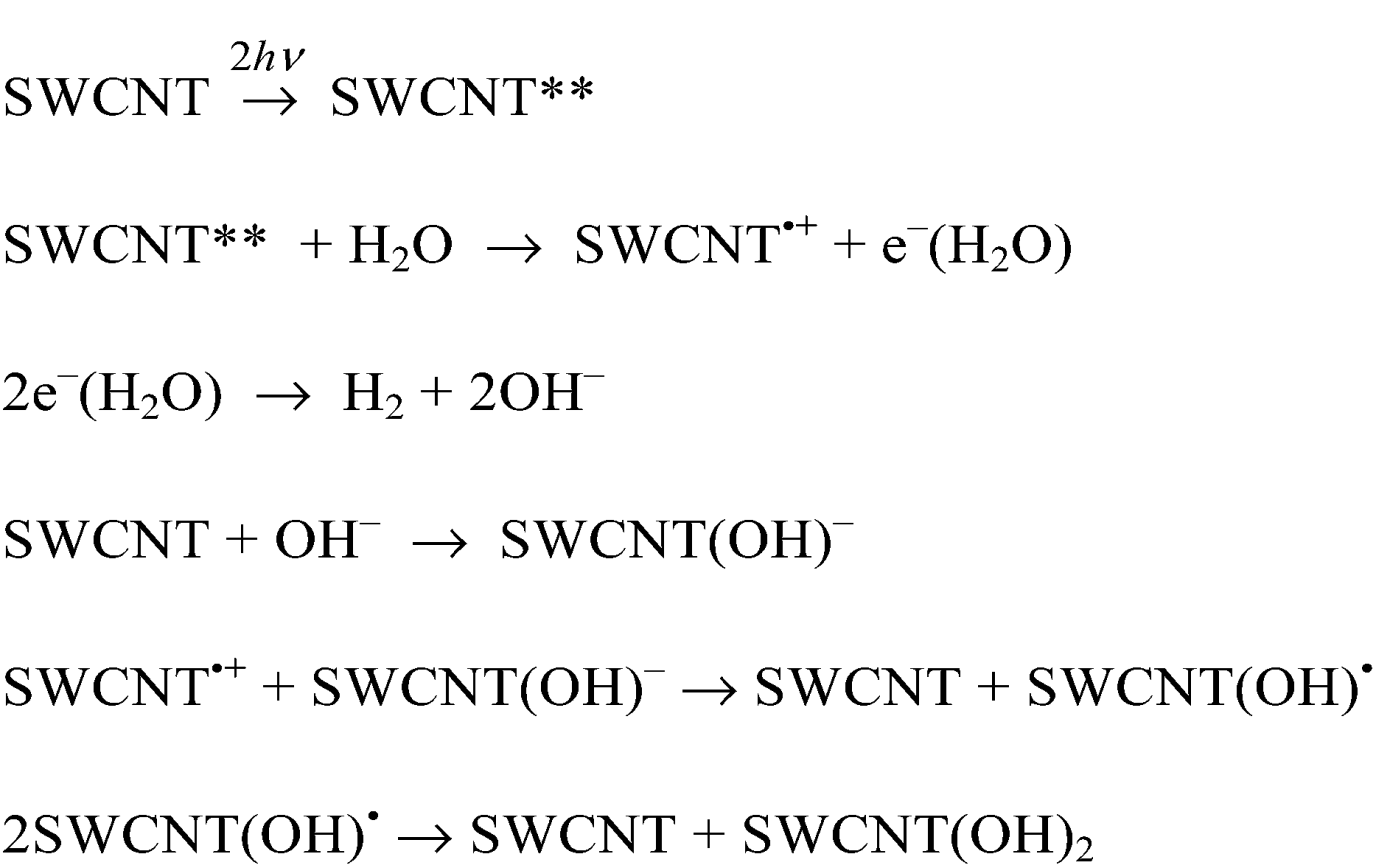


As was the case for the laser-induced H_2_ evolution in benzene, the initial rates of laser-induced H_2_ evolution in H_2_O are proportional to the fourth power of laser intensity as shown in Fig. 13. This suggests that the doubly excited SWCNT (SWCNT**) and the subsequent bimolecular reaction are involved in the H_2_ evolution. In the case of H_2_O, a solvated electron (e^–^(H_2_O)) may be produced by the reaction of SWCNT** with H_2_O as shown in Scheme 3, where the bimolecular reaction of e^–^(H_2_O) produces H_2_ and two equiv. of OH^–^ as indicated by the pulse radiolysis study of H_2_O.^46–49^ The OH^–^ may be attached to a SWCNT to produce SWCNT(OH)^–^, which is oxidized by the hole of SWCNT˙^+^ to afford SWCNT(OH)˙, which may disproportionate to yield the dihydroxylated SWCNT [SWCNT(OH)_2_], accompanied by the regeneration of the SWCNT.

## Conclusions

In conclusion, SWCNTs have been demonstrated to act as efficient photocatalysts for H_2_ evolution from benzene derivatives under laser irradiation. The TON was over 1 million based on one nanotube. The rate of H_2_ evolution increased with increasing the laser intensity, exhibiting a fourth power dependence, because hydrogen was evolved *via* the radical coupling of radical anions derived from benzene as the rate-determining step and a benzene radical anion was produced by electron transfer from the doubly excited state of a SWCNT to benzene, which requires two photons. The polymerisation of benzene induced by photogenerated C_6_H_6_˙^–^ accompanied by H_2_ evolution gains a leverage effect to increase the quantum yield of H_2_ evolution to as high as 130%, which is much larger than that expected from the four-photon process (25%). Laser-induced H_2_ evolution also occurred with SWCNTs in H_2_O, also exhibiting a fourth power dependence for the H_2_ evolution rate. In this case, H_2_ was evolved *via* the electron-transfer reduction of water by the doubly excited state of a SWCNT, and a SWCNT was oxidized to yield the dihydroxylated SWCNT. Metal-free laser-induced H_2_ evolution in aromatic compounds and H_2_O with SWCNTs found in this study paves a new way for efficient *pinpoint* hydrogen evolution, which may find various applications.

## Experimental section

### Materials

Chemicals were purchased from a commercial source and used without purification. SWCNTs (synthetic method: arc plasma jet, diameter: 1.4 nm approximate length: 1–5 μm, percentage of carbon: >99%) were obtained from Meijo Nano Carbon, Japan. The solution dispersed SWCNTs were prepared by ultrasonication (42 kHz, 125 W) for 5 min. Benzene was of spectral grade, obtained commercially and used without further purification. Deuterated benzene (C_6_D_6_, 99%) was obtained from Cambridge Isotope Laboratories, Inc., and was used as received. Benzonitrile was distilled over P_2_O_5_ *in vacuo*.^50^ Mesitylene, *p*-xylene, chlorobenzene and 1,4-dimethoxybenzene were obtained commercially and used as received. Potassium ferrioxalate used as an actinometer was prepared according to the literature and purified by recrystallisation from hot water.^39^ D_2_ gas (99.5%) was commercially obtained from Sumitomo Seika Chemicals Co., Ltd. Purification of water (18.2 MΩ cm) was performed with a Milli-Q system (Millipore, Direct-Q 3 UV).

### Reaction procedure

The photocatalytic hydrogen evolution was carried out by the following procedure. C_6_H_6_, C_6_D_6_ or benzene derivative solutions (2.5 cm^3^) containing SWCNTs (0.15 mg) in a square quartz cuvette (10 mm i.d.) sealed with a rubber septum was deaerated by bubbling with nitrogen through a stainless steel needle for 5 min. The solution was then irradiated with a Nd:YAG laser (LS2134UTF) at *λ* = 532 nm with the power of 50 mJ per pulse at room temperature. The gas in the headspace was analyzed using a Shimadzu GC-14B gas chromatograph (detector, TCD; column temperature, 50 °C; column, active carbon with 60–80 mesh particle size; carrier gas, N_2_) to quantify the evolved hydrogen. The reaction solution was analyzed by a Shimadzu GC-17A gas chromatograph and Shimadzu MS-QP5000 mass spectrometer to quantify the produced biphenyl, and HPLC [detector, UV at *λ* = 280 nm (SPD-10A, Shimadzu); column, Shim Pack VP-ODS; eluent, CH_3_CN: 0.40 mL min^–1^, water: 0.10 mL min^–1^] to qualify the generated terphenyls. Hydrogen evolved in C_6_D_6_ after 2 h laser irradiation was detected using a Shimadzu GC-8A gas chromatograph [detector, TCD; column temperature, 77 K (liquid N_2_); column, Hydro Isopack (2.0 m, 4.0 mm i.d., GTR TEC Co., Ltd.); carrier gas, Ne] to analyze H_2_, HD and D_2_ gases. In the case of measuring the laser intensity dependence, a benzene solution (2.5 cm^3^) containing SWCNTs (0.15 mg) in a square quartz cuvette (10 mm i.d.) sealed with a rubber septum was deaerated by bubbling with nitrogen through a stainless steel needle for 5 min. The solution was then irradiated using a Nd:YAG laser (LS2134UTF) at *λ* = 532 nm with the various laser intensities (18–82 mJ per pulse) at room temperature. The amount of hydrogen evolved was analyzed at 10, 20 and 30 min using a Shimadzu GC-14B gas chromatograph.

### Characterisation of SWCNTs as catalysts

Transmission electron microscopy (TEM) images of SWCNTs before and after 2 h laser irradiation (*λ* = 532 nm; 50 mJ per pulse; 10 Hz) in a benzene solution were measured using a JEOL JEM 2100 operating at 200 kV. TG data were recorded on a SII TG/DTA 7200 instrument. SWCNTs, (∼1 mg) before and after the reaction , were heated from 25 °C to 600 °C with a ramp rate of 2 °C min^–1^. A certain amount of α-Al_2_O_3_ was used as a reference for DTA measurements. Raman spectra were obtained using a JASCO NR-1800 with a 514.5 nm Ar laser. IR spectra were recorded on a JASCO FT/IR-6200, using KBr pellets.

### Quantum yield determinations

A standard actinometer (potassium ferrioxalate)^39^ was used for the quantum yield determination of hydrogen evolution from benzene with SWCNTs. A square quartz cuvette (10 mm i.d.) containing a benzene solution (2.5 cm^3^) of SWCNTs (0.15 mg) was irradiated using a Nd:YAG laser (LS2134UTF) at *λ* = 532 nm with the various laser intensities (18–82 mJ per pulse). Under the conditions of actinometry experiments, SWCNTs absorbed essentially 100% of the incident light of *λ* = 532 nm. The light intensity of the laser light of *λ* = 532 nm was determined as 6.5 × 10^–9^ einstein s^–1^ at 8.0 mJ per pulse. The photochemical reaction was monitored using a Shimadzu GC-14B gas chromatograph. The quantum yields were determined from the amount of hydrogen evolved.

### EPR measurements

The EPR spectra were measured on a JEOL X-band EPR spectrometer (JES-ME-LX) using a quartz EPR tube containing SWCNTs (24 μg) dispersed in deaerated benzene (0.20 cm^3^) by laser irradiation (*λ* = 532 nm; 40 mJ per pulse; 10 Hz) for 10 min at 77 K. The internal diameter of the EPR tube is 4.5 mm, which is small enough to fill the EPR cavity but large enough to obtain good signal-to-noise ratios during the EPR measurements. The amplitude of modulation was chosen to optimize the resolution and the signal-to-noise (*S*/*N*) ratio of the observed spectra. The *g* values were calibrated with an Mn^2+^ marker.
