# Supplementary material for: Laser-induced pinpoint hydrogen evolution from benzene and water using metal free single-walled carbon nanotubes with high quantum yields
Source: Chem Sci. 2014 Sep 9;6(1):666–74. doi: 10.1039/c4sc02269f (PMC5590240; doi:10.1039/c4sc02269f)
Supplement: Supplementary file 1 [file SC-006-C4SC02269F-s001.pdf]

Electronic Supplementary Material (ESI) for Chemical Science.
This journal is © The Royal Society of Chemistry 2014

Electronic Supplementary Information

Laser-induced pinpoint hydrogen evolution from benzene and water using metal free single-walled carbon nanotubes with high quantum yields

Kei Ohkubo, Naoki Kohno, Yusuke Yamada and Shunichi Fukuzumi*

Department of Material and Life Science, Graduate School of Engineering, Osaka University, ALCA, Japan Science and Technology Agency (JST), Suita, Osaka 565-0871, Japan

E-mail: fukuzumi@chem.eng.osaka-u.ac.jp

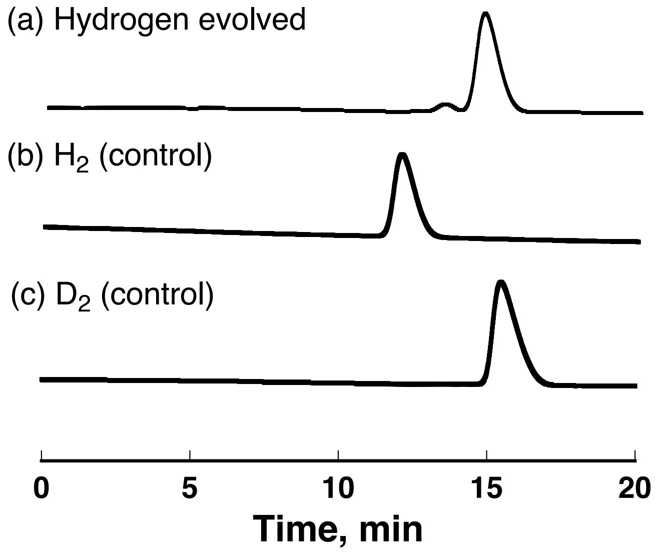

Fig. S1 GC charts of evolved hydrogen after laser light irradiation (λ = 532 nm; 50 mJ pulse^{-1}; 10 Hz) of a benzene-d_6 solution (2.5 mL) containing SWCNTs (0.15 mg) for 2 h, b) H_2 and c) D_2 as control experiments.

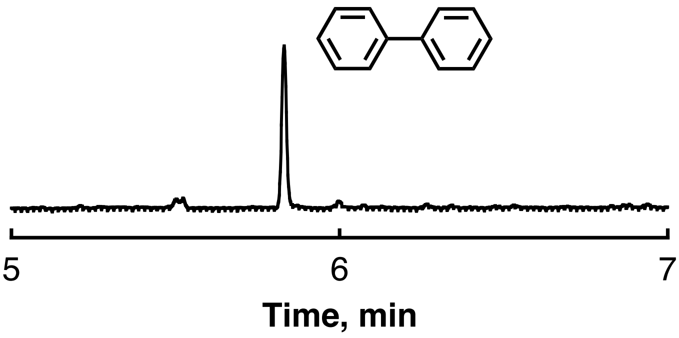

Fig. S2 GC-MS chart of benzene solution (2.5 mL) containing SWCNTs (0.15 mg) after 2 h laser irradiation ($\lambda = 532$ nm; 50 mJ pulse^{-1}; 10 Hz).

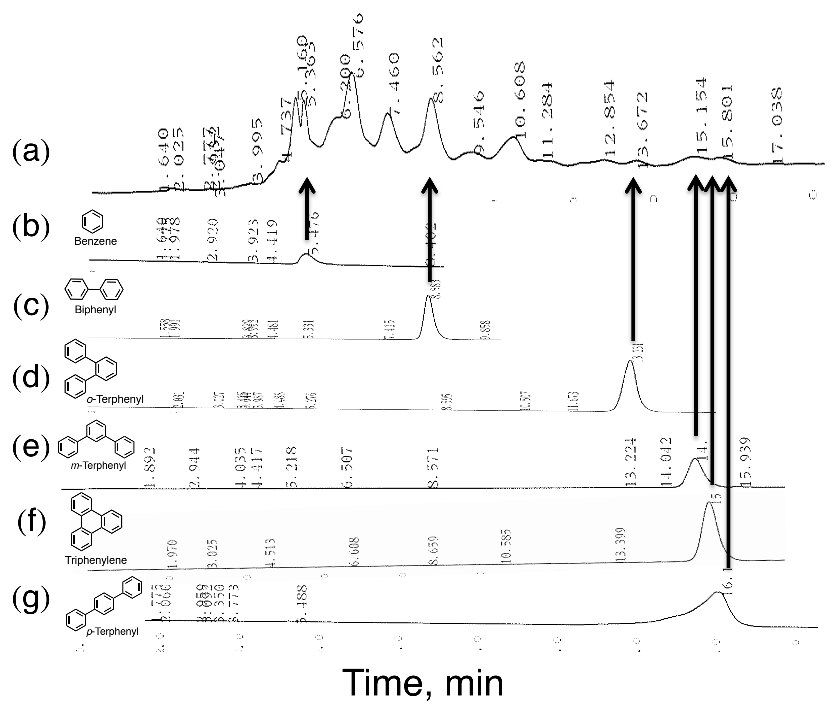

Fig. S3 HPLC charts of a) benzene solution (2.5 mL) containing SWCNTs (0.15 mg) after 2 h laser irradiation ($\lambda = 532$ nm; 50 mJ pulse^{-1}; 10 Hz), b) benzene, c) biphenyl (2 mM in CH_3CN), d) *o*-terphenyl (2 mM in CH_3CN), e) *m*-terphenyl (2 mM in CH_3CN), f) triphenylene (2 mM in CH_3CN) and g) *p*-terphenyl (2 mM in CH_3CN).

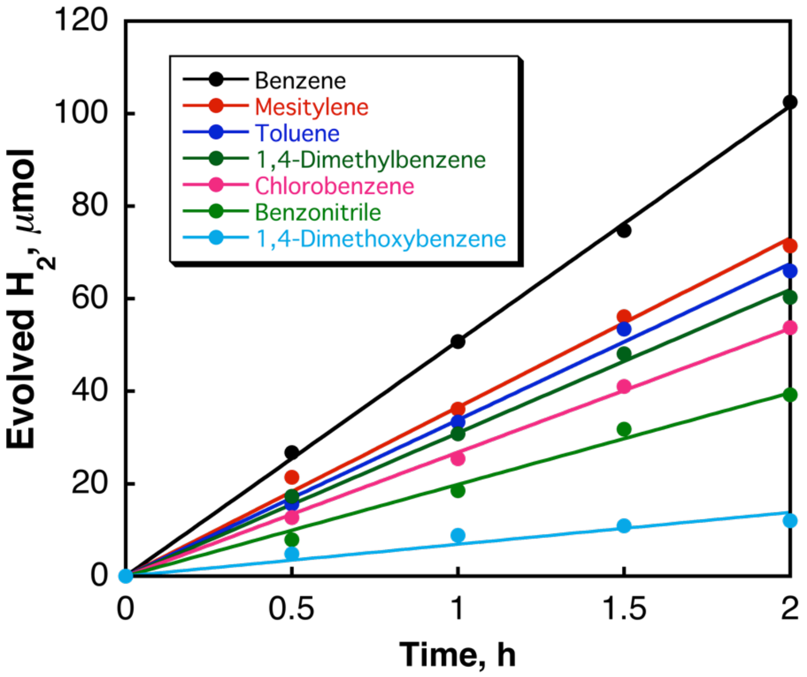

Fig. S4 Time courses of the amount of H_2 evolved under laser light irradiation (λ = 532 nm; 50 mJ pulse^{-1}; 10 pulse s^{-1}) in benzene (black), toluene (blue) mesitylene (red), *p*-xylene (green), chlorobenzene (pink), benzonitrile (light green), and 1,2-dimethoxybenzene (light blue) solutions (2.5 mL) containing SWCNTs (0.15 mg).

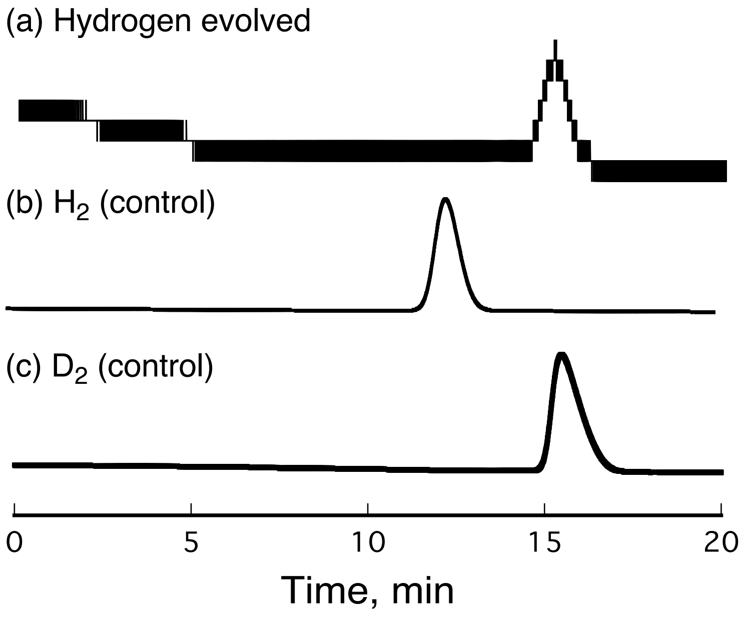

Fig. S5 GC charts of (a) evolved hydrogen after laser light irradiation (λ = 532 nm; 60 mJ pulse^{-1}; 10 Hz) of a D_2O solution (2.5 mL) containing SWCNTs (2.0 mg) for 2 h, (b) H_2 and (c) D_2 as control experiments.

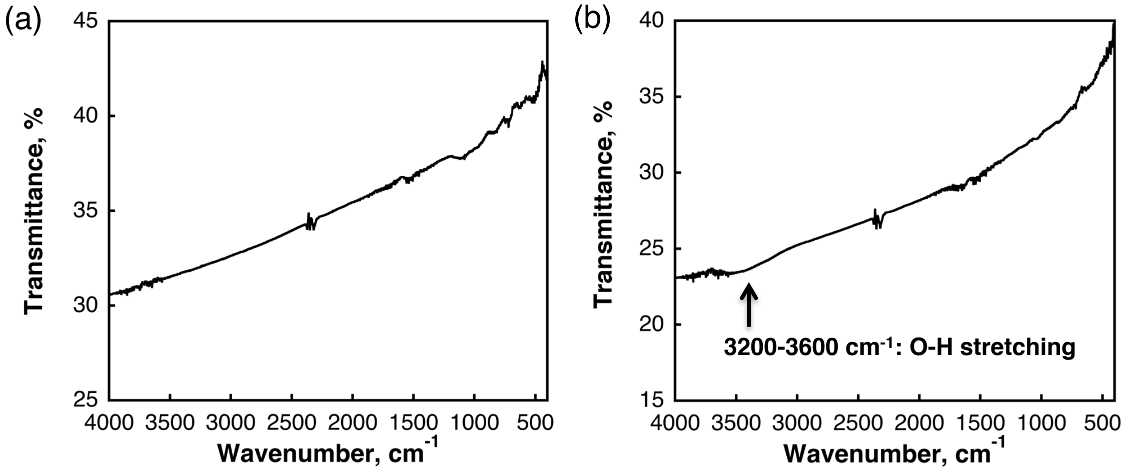

Fig. S6 IR spectra of (a) SWCNTs before laser irradiation and (b) SWCNTs obtained by laser light irradiation (λ = 532 nm; 60 mJ pulse^{-1}; 10 pulse s^{-1}) for 5 h in deaerated water (2.0 mg, 2.5 mL).

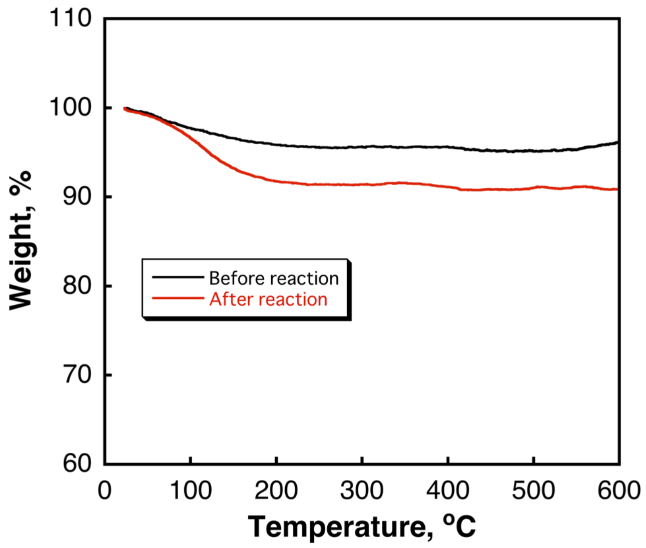

Fig. S7 TG curves of SWCNTs before and after laser irradiation (H_2 evolution) in water observed under deaerated conditions.
